# Killing wisely: precision senolytics in the age of frailty

**DOI:** 10.1101/gad.353134.125

**Published:** 2025-08-01

**Authors:** Valentin J.A. Barthet, Scott W. Lowe

**Affiliations:** 1 Cancer Biology and Genetics Program, Sloan Kettering Institute, Memorial Sloan Kettering Cancer Center, New York, New York 10065, USA;; 2 Howard Hughes Medical Institute, Memorial Sloan Kettering Cancer Center, New York, New York 10065, USA

**Keywords:** aging, senescence, senolytics

## Abstract

Cellular senescence plays a dual role in tissue biology by promoting tumor suppression and wound healing when transient but driving inflammation, fibrosis, and age-related disease when persistent. The growing recognition that senescent cell clearance can reverse these pathologies has catalyzed efforts to develop therapeutics that preferentially kill senescent cells (also known as “senolytics”). However, clinical translation from bench to bedside remains challenging due to senescent state heterogeneity, limited biomarkers, off-target toxicities, and the frailty of aged patients. Small molecule senolytics, although promising, often lack defined mechanisms of action and pose safety concerns that may constrain their use in older adults. Emerging precision approaches, including those that exploit surface markers and leverage engineered immune therapies, offer a rational and potentially more selective path forward. Here we highlight recent advances in senescence profiling and targeted clearance strategies, emphasizing the need for therapies designed with both biological complexity and the needs of aging populations in mind.

## Abstract (precis)

In this Outlook, Barthet and Lowe discuss recent advances in characterizing cellular senescence, highlighting the duality of senescence programs—being both beneficial and pathological—in a context-dependent manner. They also explore the promise and limitations of senolytic strategies for the precise and selective targeting of senescent cells, with wide-ranging clinical implications for therapeutic interventions in aging and disease.

Cellular senescence is a program that triggers proliferative arrest and elicits a secretory program that reshapes the tissue microenvironment. Although the induction of senescence can limit oncogenic transformation and aid in wound healing, the aberrant accumulation of senescent cells in damaged tissues promotes chronic inflammation, fibrosis, and immune dysfunction (Kuehnemann and Wiley 2024). These dichotomous roles underscore the therapeutic potential and inherent complexity of targeting senescent cells in vivo.

## Senescence: beneficial by design, pathological by persistence

Cellular senescence was first characterized in fibroblasts undergoing replicative exhaustion in culture (Hayflick and Moorhead 1961), where it is characterized by irreversible cell cycle arrest related to the upregulation of tumors suppressors such as p16 and p53, leading to downregulation of proliferative genes and the increased transcription of factors linked to the “senescence-associated secretory phenotype (SASP)” and cell surface proteins that sense environmental signals (Kumari and Jat 2021; Chen et al. 2023). SASP factors include proinflammatory cytokines, chemokines, and matrix-remodeling enzymes (Coppé et al. 2010), whereas surfaceome components include immune modulatory factors such as NK cell ligands and the interferon γ receptor (Sagiv et al. 2016; Chen et al. 2023). It seems likely that the latter features contribute to the diversity of senescence biology by influencing whether senescent cells are cleared by the immune system or persist.

In young people, evidence suggests that senescence has a beneficial effect by maintaining tissue integrity and preventing the early emergence of cancers. However, in the elderly, senescent cells accumulate across tissues, perhaps due to declining immune surveillance (Franceschi et al. 2018). These senescent-like cells are clearly detrimental, contributing to organ decline by altering stem cell niches and producing fibrosis, and create immunosuppressive microenvironments permissive to cancer progression (Parrinello et al. 2005). Accordingly, clearing senescent cells genetically or pharmacologically can delay or reverse features of aging and disease in mice (Baker et al. 2011; Xu et al. 2018). Still, precisely who these cells are and how they drive pathology remain unclear.

## Senescence is not one cell fits all

The definitions of senescence are ambiguous, but traditional “hallmarks” include increased β-galactosidase activity, upregulation of p16, appearance of DNA damage foci, and the SASP (Ogrodnik et al. 2024). Unlike functionally defined cell states such as “stem cells,” which are operationally characterized by self-renewal and differentiation capacity, senescence is defined largely by consensus around a set of context-dependent markers rather than a clear biological function (Ogrodnik et al. 2024). Still, it is important to remember that, although we often develop artificial in vitro models to approximate physiological processes observed in vivo, the trajectory of senescence research has proceeded in reverse, with in vitro phenotypes used to define senescent states in vivo. Therefore, it should not be surprising that it has been difficult to precisely pinpoint and study senescent cells in tissue. This in vitro origin continues to shape prevailing assumptions in the field, highlighting a need for better in vivo definitions and more precise phenotyping of distinct senescent-like states.

What is clear, however, is that cell states displaying senescence features are molecularly diverse, with overlapping but distinct features based on cell type, senescence inducer, and organ context. Indeed, in-depth molecular characterization of various senescence contexts has uncovered distinct senescent subtypes with varying SASP composition, metabolic adaptations, and immune interactions (Coppé et al. 2010; Baker et al. 2011; Zhu et al. 2015; Yosef et al. 2016; Childs et al. 2017; Xu et al. 2018). For example, senescent fibroblasts differ markedly in their transcriptional and secretory profiles depending on whether senescence was induced by irradiation, oncogene activation, or injury (Hernandez-Segura et al. 2017).

Features associated with senescent state heterogeneity are increasingly recognized as influencing tissue biology in diverse ways, including the capacity to elicit immune surveillance. For example, recent work demonstrated that genetic ablation of p16-expressing macrophages versus endothelial cells had opposing effects on liver fibrosis in mice, highlighting that both beneficial and detrimental senescent states can coexist within a single pathological setting (Zhao et al. 2024). This complexity complicates the interpretation of preclinical models and the therapeutic application of senolytic agents. Notably, compounds deemed senolytic in one context may lack efficacy or exhibit toxicity in others, as exemplified by the BCL2 family inhibitor navitoclax (Wang et al. 2017). More broadly, indiscriminate targeting of senescent cells may be undesirable. Thus, effective therapeutic strategies will require a nuanced understanding of senescent cell identity and those senescent-like states that drive pathology.

## Mark the cell, make the kill: surfaceome-guided senolysis

Despite their promise and ease of use, small molecule senolytics face notable limitations. These agents depend on molecular vulnerabilities, such as reliance on antiapoptotic regulators, that are not consistently defined across senescent cell states. In many cases, mechanisms of action remain unclear and predictive biomarkers of sensitivity or resistance are lacking, complicating patient stratification and response assessment. As a result, it is challenging to determine whether any observed benefit in patients is due to senescent cell clearance.

An emerging alternative involves targeting cell surface proteins that are upregulated in senescent cells, leveraging antibody-based therapeutic modalities used broadly in cancer therapy and increasingly beyond. A major advantage of these approaches is that both efficacy and on-target toxicities can be readily predicted based on expression of the target protein. In contrast to cancer, where loss of a nonessential target can promote resistance, senescent cells are postmitotic; those that escape targeting are unlikely to proliferate and restore disease.

One such target is the urokinase plasminogen activator receptor (uPAR), a GPI-anchored protein involved in wound healing and tissue remodeling (Rømer et al. 1996). uPAR is minimally expressed in vital organs but is consistently upregulated across senescent states associated with fibrosis and in aged tissues (Amor et al. 2020, 2024). uPAR-targeting CAR T cells can eliminate senescent-like cells, thereby resolving tissue fibrosis as well as improving age-related fitness loss, metabolic syndrome, and inflammaging (Amor et al. 2020, 2024). Other studies have used bispecific T-cell engagers targeting uPAR and immune checkpoint proteins (Chu et al. 2023), as well as strategies to enhance natural killer (NK) cell activity, including boosting recognition of NKG2D ligands naturally induced on senescent cells (Deng et al. 2024). As surfaceome profiling expands, such approaches, including dual-targeted and logic-gated designs, offer a path toward precise, tissue-selective senescence-directed therapies.

## From promise to precision: making senotherapeutics safe

The convergence of single-cell profiling, synthetic biology, and senescence surfaceome mapping is reshaping our understanding of senescent cell diversity and may inform the next generation of targeted therapies. Rather than applying “one size fits all” senolytics, future strategies must selectively eliminate pathogenic senescent cell subtypes within the relevant tissue and disease context. This need is especially acute for therapies destined to treat elderly patients, where therapeutic benefit must be weighed against age-related organ decline, tissue repair, and altered pharmacokinetics. Indeed, potent senolytic agents such as navitoclax cause dose-limiting thrombocytopenia (Schoenwaelder et al. 2011), a toxicity that is intolerable in frail patient populations despite its remarkable activity in mice.

Looking forward, clinical translation of senolytic therapies should be based on a rational understanding of senescence mechanisms, functional studies to confirm that clearance does not impair tissue regeneration, and dosing of selective strategies that preserve beneficial senescence while eliminating its pathogenic forms. We believe that therapies targeting cell surface proteins (e.g., CAR T cells, bispecific engagers, and NK cell-directed approaches) provide mechanistically guided therapeutics that should facilitate patient stratification and will enable validation of the “senolytic” concept in patients. By refining the precision of senescent cell clearance and prioritizing safety in the aged, these emerging strategies have the potential to transform the treatment of senescence-associated diseases.

## A call to the field

As senescence-modulating therapies inch closer to the clinic, the field must confront two central truths. First, senescence is not a uniform entity: It is a collection of diverse, context-dependent cell states with distinct biological consequences. Rational therapeutic design demands an equally nuanced approach, one that matches intervention to the senescent subtype, tissue context, and disease setting. Second, and just as critically, senotherapies must be developed for those who need them most: elderly patients. This requires an age-informed framework that considers altered pharmacodynamics, diminished regenerative capacity, and heightened susceptibility to toxicity. In this light, specificity is not a luxury, it is a prerequisite. The era of blunt senolytics must give way to one of precision senotherapies: programmable, context-aware interventions capable of eliminating pathogenic senescent cells while preserving those with reparative or homeostatic functions. The central question is no longer whether we can kill senescent cells but whether we can do so wisely and safely for those living at the intersection of aging and disease.
